# Complete Genome Sequence of a *Propionibacterium acnes* Isolate from a Sarcoidosis Patient

**DOI:** 10.1128/genomeA.00016-12

**Published:** 2013-01-15

**Authors:** Kana Minegishi, Chihiro Aikawa, Asuka Furukawa, Takayasu Watanabe, Tsubasa Nakano, Yoshitoshi Ogura, Yoshiyuki Ohtsubo, Ken Kurokawa, Tetsuya Hayashi, Fumito Maruyama, Ichiro Nakagawa, Yoshinobu Eishi

**Affiliations:** a Department of Human Pathology; b Section of Bacterial Pathogenesis; c Graduate School of Medical and Dental Sciences, Tokyo Medical and Dental University, Tokyo, Japan; Division of Microbiology, Department of Infectious Diseases, Faculty of Medicine; d Division of Bioenvironmental Science, Frontier Science Research Centre, University of Miyazaki, Miyazaki, Japan; e Department of Environmental Life Sciences, Graduate School of Life Sciences, Tohoku University, Sendai, Japan; f Department of Biological Information, Graduate School of Bioscience and Biotechnology, Tokyo Institute of Technology, Kanagawa, Japan

## Abstract

*Propionibacterium acnes* is a human skin commensal that resides preferentially within sebaceous follicles and is the only microorganism that has been isolated from sarcoid lesions. We report the complete genome sequence of *P. acnes*, which was isolated from a Japanese patient with sarcoidosis.

## GENOME ANNOUNCEMENT

*Propionibacterium acnes* is generally considered to be a commensal organism on human skin and has been implicated in various infections, including acne vulgaris, endocarditis, osteomyelitis, and prostate cancer (1–4). *P. acnes* is also the only microorganism that has been isolated from sarcoid lesions by bacterial culture (5). We recently demonstrated an etiologic link between sarcoidosis and this bacterium by detecting *P. acnes* in formalin-fixed paraffin-embedded tissue sections from patients with sarcoidosis using novel *P. acnes-*specific monoclonal antibodies (6). However, how *P. acnes* causes sarcoidosis is currently unknown. The complete genome sequences might help to reveal specific *P. acnes* genes related to development of sarcoidosis, but so far, only the genomes of *P. acnes* strains derived from patients with diseases other than sarcoidosis have been fully sequenced (7–11). We describe the first complete genome sequence of *P. acnes* isolated from a Japanese patient with sarcoidosis.

The complete genome sequence of *P. acnes* strain C1 was determined using a combination of 454 GS Junior (Roche; 230,769,391-bp sequences, 92-fold coverage) and Genome Analyzer IIx (Illumina; 138,753,576-bp sequences, 55-fold coverage) sequencing platforms. Assembly was performed using Newbler. Gaps between adjacent contigs were closed by sequencing PCR amplicons from genomic DNA. Automatic annotation of the genome was performed using the NCBI Prokaryotic Genomes Automatic Annotation Pipeline (PGAAP) (http://www.ncbi.nlm.nih.gov/genomes/static/Pipeline.html). Nontranslated genes were predicted using tRNAscan-SE (12), RNAmmer (13), and Rfam (14).

The genome of *P. acnes* strain C1 contained a single circular chromosome (2,519,002 bp; 60.06% G+C content). The chromosome contained 2,359 coding DNA sequences (CDSs), nine rRNAs, and 45 tRNA sequences. The all-to-all BlastP analysis with protein sequences of the two sequenced strains, ATCC 11828 and SK137, showed that *P. acnes* C1 possessed 132 strain-specific CDSs. Several insertion sequence elements on the C1 genome detected by ISfinder (15) showed high homology to proteins of different species of the same genus, such as *Propionibacterium freudenreichii*, suggesting that *P. acnes* strain C1 may have acquired these CDSs through horizontal gene transfer from these species. Approximately half of the strain-specific CDSs were annotated as hypothetical proteins. Dot plot analysis comparison with ATCC 11828 and SK137 genome sequences indicated that genome rearrangements occurred between C1 and ATCC 11828 along the replication axis but not between C1 and SK137. This may be the result of the fact that ATCC 11828 belongs to the type II division, whereas C1 and SK137 belong to the type I division according to gene sequence comparison of the *recA* and *tly* genes (16). There remain many questions regarding how this bacterium is associated with development of sarcoidosis, but the genome information on *P. acnes* C1 could promote further genomic analysis and lead to the exact pathogenetic mechanism of sarcoidosis by *P. acnes* infection.

### Nucleotide sequence accession number. 

The completed genome sequence of *P. acnes* C1 was deposited in the DDBJ/EMBL/GenBank database under accession number CP003877.
